# Validating internal controls for quantitative plant gene expression studies

**DOI:** 10.1186/1471-2229-4-14

**Published:** 2004-08-18

**Authors:** Amy M Brunner, Igor A Yakovlev, Steven H Strauss

**Affiliations:** 1 Department of Forest Science, Oregon State University, Corvallis, OR 97331-5752, USA; 2 Skogforsk/ Norwegian Forest Research Institute, Hogskoleveien 12, N-1432 As, Norway

## Abstract

**Background:**

Real-time reverse transcription PCR (RT-PCR) has greatly improved the ease and sensitivity of quantitative gene expression studies. However, accurate measurement of gene expression with this method relies on the choice of a valid reference for data normalization. Studies rarely verify that gene expression levels for reference genes are adequately consistent among the samples used, nor compare alternative genes to assess which are most reliable for the experimental conditions analyzed.

**Results:**

Using real-time RT-PCR to study the expression of 10 poplar (genus *Populus*) housekeeping genes, we demonstrate a simple method for determining the degree of stability of gene expression over a set of experimental conditions. Based on a traditional method for analyzing the stability of varieties in plant breeding, it defines measures of gene expression stability from analysis of variance (ANOVA) and linear regression. We found that the potential internal control genes differed widely in their expression stability over the different tissues, developmental stages and environmental conditions studied.

**Conclusion:**

Our results support that quantitative comparisons of candidate reference genes are an important part of real-time RT-PCR studies that seek to precisely evaluate variation in gene expression. The method we demonstrated facilitates statistical and graphical evaluation of gene expression stability. Selection of the best reference gene for a given set of experimental conditions should enable detection of biologically significant changes in gene expression that are too small to be revealed by less precise methods, or when highly variable reference genes are unknowingly used in real-time RT-PCR experiments.

## Background

For many years the vast majority of gene expression studies have employed non-quantitative or semi-quantitative RNA gel blots and RT-PCR analysis. Real-time PCR technology has removed many of the difficulties associated with quantitative gene expression studies [1], and real-time quantitative RT-PCR (qRT-PCR) is rapidly being adopted as a standard method for in-depth expression studies, including studies of alternative splicing, verification of microarray expression results, and molecular diagnostics [2-5].

Real-time qRT-PCR offers a robust means for precisely quantifying changes in gene expression over a wide dynamic range. It is also applicable to experiments where RNA amounts are limiting, such as for micro-dissected tissues. However, selection of an appropriate normalization method is crucial for reliable quantitative gene expression results [1,6]. The purpose of normalization is to correct for non-specific variation, such as differences in RNA quantity and quality, which can affect efficiencies of the RT and PCR reactions.

Normalization to total RNA content poses a number of problems. It is difficult to quantify small amounts of RNA, and variation in RT and PCR reaction efficiencies are not accounted for by this method. Similarly, normalization to an external RNA standard is problematic due to RNA instability. The most commonly used method is relative quantitation, whereby gene expression level is normalized to that of an internal reference gene. While this avoids the problems and limitations of absolute quantitation, selection of a proper internal control–gene expressed at a nearly constant level in all tissue samples being investigated–is required. Failure to use an appropriate control gene may result in biased gene expression profiles, as well as low precision. The consequences may be that only gross changes in expression level are declared statistically significant, or that patterns of expression are erroneously characterized.

Until recently, internal controls (often referred to as housekeeping or maintenance genes), were selected based on stability of expression in qualitative studies (e.g., visual examination of RNA gel-blots), via low-sensitivity assays such as densitometry of hybridized blots, or via semi-quantitative RT-PCR. None of these will be adequate for identifying reliable internal controls for real-time qRT-PCR. For example, expression profiling via real-time qRT-PCR of 10 commonly used human internal control genes revealed different degrees and patterns of expression among 13 tissue types, and no single gene was a suitable universal control for all tissue types [7]. Although *18S *rRNA is frequently used as an internal control, it is far from ideal. It requires the use of total RNA and random primers for the RT reaction, and is expressed at very high levels; some means for attenuating *18S *expression might be needed when weakly expressed genes are studied. In addition, there can be imbalances in rRNA and mRNA fractions between different samples, and *18S *is not always expressed at a constant level in all conditions [1]. Finally, *18S *expression levels appear to be affected to a lesser extent by partial RNA degradation than are mRNA expression levels [8].

Studies in mammalian and microbial systems, where real-time qRT-PCR has been most extensively applied to date, have begun to include evaluations of various housekeeping genes for normalization [7-11]. Vandesompele et al. [7] recognized the importance of using statistical approaches to selecting the best internal controls for a given set of samples, and developed a procedure to select internal controls based on the mean pairwise variation of a gene from all other tested control genes. The adoption of real-time qRT-PCR methodology is somewhat reminiscent of the introduction of cDNA expression microarrays in that initial microarray studies did not identify differentially expressed genes by a statistical method, but by an arbitrary cut-off value of fold-change [12]. Similarly, the first real-time qRT-PCR studies have generally normalized expression levels to an internal control that is assumed to be valid rather than one that has been shown to be valid by statistical analysis of data. More rigorous methods will be needed as qRT-PCR is increasingly applied to study of regulatory genes, and for verifying patterns observed in microarray experiments.

In this study, we used real-time qRT-PCR to examine the expression of 10 housekeeping genes in a diversity of poplar (*Populus trichocarpa *× *P. deltoides*, cottonwood hybrid) tissues collected at different developmental stages, and at different times of the year. The goal of our studies was to detect changes associated with seasonal development and tree aging for several regulatory genes. We therefore undertook a study to compare the stability of several potential control genes. We found that the genes tested exhibited very different degrees of variation in expression among tissue samples, and that a statistical and graphical method helped us to select the genes best suited for the developmental studies we were conducting. This approach, which is very similar to a classical method used by plant breeders to assess the relative stability in yield of different varieties [13], can be applied to any gene or set of tissues to identify the most stable internal controls.

## Results and Discussion

### Expression profiling of poplar housekeeping genes

Ten housekeeping genes that represent different functional classes and gene families were chosen for study. These include ubiquitins, actins, tubulins, cytosolic cyclophilin (peptidyl-prolyl isomerase), translational initiation factor, elongation factor, and rRNA. Searches of the literature revealed that members of all classes have been used as internal controls for studies of plant gene expression using RNA gel blots or RT-PCR assays. Poplar genes belonging to these gene families were identified via searches of the EST database (Table 2). The expression level of these genes was determined in eight tissue samples (Table 1) collected over a seven month period from mature female poplar trees growing in plantations in Oregon, USA.

Within a single experiment, aliquots of the same cDNA synthesis reaction were used for real-time PCR amplification of each of the 10 genes and all gene primer and cDNA combinations were amplified in triplicate in a single PCR run. The entire experiment was then repeated a second time and results combined for statistical analysis. Quantitation via real-time PCR is based on cycle threshold (C_T_). C_T _is the cycle at which a significant increase in amount of PCR product (measured by increase in fluorescence) occurs, generally the middle of the exponential phase of amplification.

Mean C_T _values (average of both experiments) for each gene are given in Table 3. We had previously used *18S *as an internal control for expression studies using these and other tissue samples and had noticed that *18S *C_T _values sometimes varied considerably (data not shown). This may have been largely due to the high abundance of *18S *transcripts. Use of *18S *as an internal control for studies of genes expressed at relatively low levels required additional dilution of the cDNA templates for 18S amplification relative to the gene being studied. In the present study, the amount of cDNA was the same for all PCR reactions, but *18S *primer concentrations were 50 nM, while all other gene primer concentrations were 600 nM. As expected, *18S *was the most abundant (lowest C_T_) housekeeping transcript; *TUA *was the least abundant.

### Statistical analysis of stability of gene expression level

**Figure 1:**
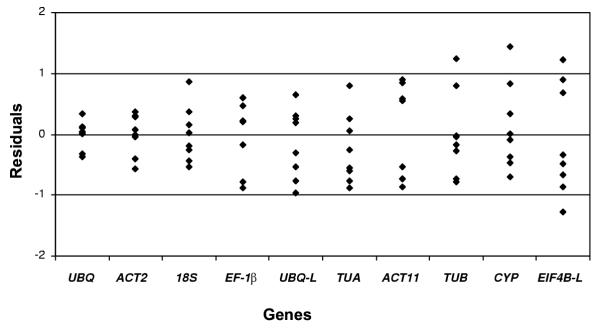
**Scatterplot of residuals after regression of tissue means from each experiment on overall mean for all genes. **Genes were ordered based on absolute value of mean residuals (increasing from left to right).

We used single-factor ANOVA and linear regression analyses of C_T _values to examine variation among tissues and RT-PCR experiments. Examination of the distribution of the residual values from ANOVA indicated that assumptions concerning homogeneity of variance and normality of data were adequately met (data not shown). The ANOVA F-test of differences among tissues indicated that five of the genes showed significant variation in expression among the tissue samples. The degree of residual variation, as reflected in the mean square error (MSE), residuals, or coefficient of variation (CV), varied widely. Four genes had CVs below 5%, and two had CVs at or above 25% (Table 3). The mean absolute value of the residuals (Fig. 1) varied 4.2-fold, from a level of 0.72 for ACT11 to 0.17 for UBQ. To test whether this variation could be due to chance alone, we tested the variation in size of residuals via Levene's test (Levene 1960). The variation among genes was highly significant (P < 0.004), and the difference in residuals between ACT11 and UBQ was also significant based on Tukey's Studentized Range Test and the Bonferroni t-test at the 5% confidence level.

**Figure 2:**
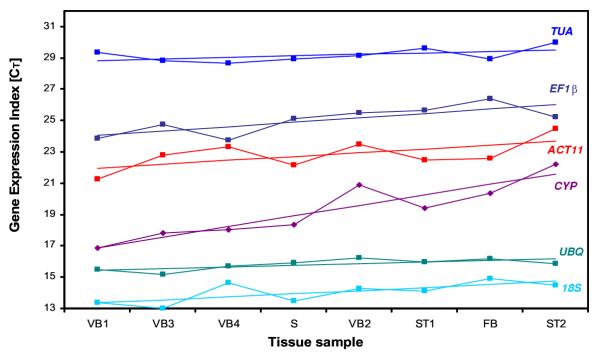
**Regression lines for several genes showing predicted regression lines and actual means over both experiments. **The most stable and consistent control genes would have the lowest slope and closest fit to the regression line. *UBQ *(second from bottom) had the highest and *TUA *(top) the second highest stability indices in this experiment. *CYP *(third from bottom) had the lowest stability index. See Table 1 for descriptions of tissue samples.

The mean expression level for each gene in each tissue sample was regressed against the overall means for the different tissue samples. This overall mean provides an index of RNA quality and quantity for that tissue sample, much as means over test sites provide an index of site fertility in yield trials [13]. The slope provides an estimate of the degree to which the gene is sensitive to general expression-promoting conditions, and the residuals (deviation from regression prediction) and mean squared residuals (MS-Reg) estimate the degree to which expression of a gene varies unpredictably after linear effects are removed. The residual variation after regression was substantial (Figure 2); MS-Reg varied approximately 14-fold (Table 3). Assuming that both constancy over tissues (low slope) and high predictability (low CV) are desired, we created a stability index as the product of slope and CV. The genes with the lowest stability index will usually provide the best controls. In this study, *UBQ *had the lowest stability index, a result of both a very low slope and very low CV.

### Selection of internal controls

In addition to constancy of expression level, the expression level of an internal control compared to that of the genes being analyzed might be important to consider in certain cases. In our study, two of the most stably expressed genes represented opposite ends of the spectrum. *UBQ *is highly expressed (mean C_T _= 15.8), whereas *TUA *is expressed at a much lower level (mean C_T _= 28.9) (Table 3). For the samples we tested, the high stability of *UBQ *and *TUA *expression indicate that use of either as a single internal control gene is appropriate. However, for some studies, no single gene may be adequate. In these cases, a method for normalization to two or more of the most stable internal control genes identified might be necessary. For example, normalizing to the geometric mean of selected internal control genes [7]. A potential strategy to avoid the additional expense and labor of using multiple internal control genes is to design a PCR primer pair that will amplify two or more members of a control gene family, whose combined expression level may exhibit the desired expression level and stability.

Our primers were designed based on a limited EST set that likely did not include all family members, and ESTs vary in sequence quality. Thus, primers could have amplified more than one family member or primer mismatches due to EST sequence errors could have lowered PCR efficiency. Although gel and real-time PCR dissociation curve analyses did not indicate that multiple genes were amplified with our primer sets, these analyses might not detect multiple amplicons from different family members that are the same size and have the same PCR efficiency. As discussed above this is not necessarily a detriment–amplification of multiple family members might result in a more stable internal control than single gene amplification. In addition, the upcoming release of a large poplar unigene set and annotated genome sequence [14] will improve gene selection and primer design capabilities.

## Conclusions

Using ANOVA and linear regression analysis, we demonstrated that levels of expression stability among a number of potential control genes can vary widely, and that it is not difficult, costly or labor-intensive to test a number of genes. Moreover, such validation tests might have the additional benefit of revealing technical problems, such as excessive variability in RT and PCR efficiency due to RNA quality or inconsistent pipetting.

For some experiments, choice of an internal control is straightforward. For example, a number of housekeeping genes should be satisfactory controls for comparisons of transgene expression level in the same tissue type from different transgenic lines grown under identical conditions. However, this is not the case for studies that compare gene expression among different tissue or cell types, at different developmental stages, or under different environmental conditions, as were represented in our study of trees in field environments over a period of seven months. For such studies, internal controls should be carefully tested and validated. Statistical confirmation of internal controls for qRT-PCR should enable previously indiscernible small changes in expression level to be reliability detected.

## Methods

### Tissue collection and RNA extraction

Tissues were collected from five or six year-old ramets (genetically identical trees) of a single female poplar hybrid clone (*P. trichocarpa *× *P. deltoidies*) over a seven-month period in 2001 (Table 1). The trees had been growing in commercial plantations in the Columbia River basin northwest of Portland, Oregon USA. Bud scales were removed and tissues were frozen in liquid N_2 _and stored at -80°C until RNA extraction. Total RNA was isolated using the RNeasy mini kit (Qiagen, Valencia, CA, USA) with modifications. Tissues (0.2 g) were ground to a fine powder with mortar and pestle in liquid N_2_. The powder was added to a tube containing 1 ml of RNeasy RLT buffer and 0.01 g soluble polyvinylpyrrolidone (PVP-40; Sigma, St. Louis, MO, USA), and homogenized using a polytron for approximately 30 sec. Four volumes of 5 M Potassium acetate, pH 6.5 was added to the homogenate, the mixture was incubated on ice for 15 min, and the precipitate removed by a 15 min centrifugation (12,000 rpm) at 4°C. Supernatant was transferred to two 1.5 ml microcentrifuge tubes and 0.5 volume of 100% EtOH was added. Samples were transferred to RNeasy mini columns and the remaining steps were as directed by the manufacturer's instructions for plant RNA isolation (steps 6–11). RNA was quantified using spectrophotometric OD_260 _measurements and quality was assesed by OD_260_/ OD _280 _ratios and by electrophoresis on 1% formaldehyde agarose gels followed by ethidium bromide staining. RNAs were stored at -80°C.

### Selection of poplar sequences and PCR primer design

To identify poplar homologs of genes commonly used as controls for plant gene expression studies, we queried poplar EST databases with Arabidopsis protein sequences using TBLASTN [15]. Selected poplar ESTs were then used to query the Arabidopsis protein database using BLASTX (Table 2). Primers were designed using Primer3 software [16] or Primer Express (Applied Biosystems, Foster City, CA, USA) with melting temperatures of 59–60°C. By comparison to related poplar EST sequences, primers were designed to be as specific as possible for the selected gene family member. All primer pairs were initially tested via standard RT-PCR using the same conditions as described below for real-time RT-PCR. Amplification of single products of expected size was verified by electrophoresis on 3% agarose-1000 (Invitrogen, Carlsbad, CA, USA) and ethidium bromide staining.

### Real-time RT-PCR

Contaminating DNA was removed from RNA samples using the DNA-Free kit (Ambion, Austin, TX, USA) according to the manufacturer's protocol, and two-step real-time RT-PCR performed. cDNA was synthesized from 5 μg of RNA using the SuperScript first-strand synthesis system for RT-PCR (Invitrogen) with random hexamer primers according to the manufacturer's instructions, except that the initial 65°C denaturation step was omitted. The cDNAs were diluted 1:5 with nuclease-free water. Aliquots of the same cDNA sample were used with all primer sets for real-time PCR, and amplification reactions with all primer sets were performed in the same PCR run. Reactions were done in a 25 μl volume containing 600 nM of each primer, 6.5 μl of cDNA sample (≈320 ng of input RNA) and 1X SYBR Green PCR master mix (Applied Biosystems). For 18S amplification, primer concentration was 50 nm. Real-time PCR was performed on the ABI Prism 7700 Sequence Detection System (Applied Biosystems) in a 96-well reaction plate using the parameters recommended by the manufacturer (2 min. at 50°C, 10 min. at 95°C and 40 cycles of 95°C for 15 sec and 60°C for 1 min.). Each PCR reaction was performed in triplicate and no-template controls were included. Specificity of the amplifications was verified at the end of the PCR run using ABI Prism Dissociation Curve Analysis Software. The entire experiment, including both the RT and real-time PCR steps, was repeated, giving a total of two experimental replications.

### Statistical analyses

Results (C_T _values) from the ABI PRISM 7700 Sequence Detection System were analyzed in Microsoft Excel. Single factor ANOVA and regression analysis using the least squares method were performed using the Excel Analysis ToolPak. Assumptions concerning homogeneity of variance and normality were evaluated from inspection of residuals (the difference between an observed value and overall mean for all genes) from the ANOVA (Fig. 1). The level and significance of the difference between gene expression levels in different samples were evaluated by Fisher's F statistic [F = between-tissue-sample mean square / error mean square] assuming the three replicate PCR reactions approximated variance between fully independent observations. Other statistics are as defined in Table 3.

The general procedure for data analysis to compare genes for use as internal controls was:

1) Generate data from multiple analyses of gene expression via quantitative RT PCR that can be assumed to be statistically independent (or nearly so), including from multiple independent samples that bracket the experimental conditions of interest.

2) Conduct ANOVA to examine the extent of variation among samples, and (optionally) test their significance using appropriate F-ratios. Examine plot of residuals vs. mean expression level, or use a statistical test, to check normality of data.

3) Genes showing high variance among tissues in ANOVA, especially if accompanied by large mean square errors (or coefficients of variation), are to be avoided as controls.

4) Calculate mean expression level over all genes studied for each sample type as an index of both experimental and biological conditions that promote high levels of measured expression. Use of a large number of genes and tissue samples (e.g., at least five, and preferably many more) are desirable where estimates of stability are to be compared between studies.

5) Regress mean expression level for each gene in each sample type over the mean for the sample type. The estimated slope and mean square residual (deviation from regression prediction) provide estimates of the degree to which the gene is sensitive to general expression-promoting conditions (slope) and whose expression continues to be difficult to predict (residual).

6) Assuming that both constancy over sample types (low slope) and high predictability (low coefficient of variation) are desirable, a stability index can be created as their product (or via other mathematical means), and the gene with the lowest value chosen.

7) Alternatively, visually inspect regression and residual plots to select genes that would be most suitable as controls for specific sets of experimental conditions.

## Authors' contributions

AMB developed the molecular methods, participated in design of the study, and drafted the manuscript. IAY designed primers, carried out the real time RT-PCR experiments, performed the statistical analysis and drafted the manuscript. SHS conceived the analysis, and participated in its design. All authors read, helped to edit, and approved the final manuscript.

## Figures and Tables

**Table 1:** Poplar tissues used for gene expression studies.

**Sample name**	**Collection date (2001)**	**Tissue description**
VB1	March 20	Overwintered terminal vegetative buds
VB2	April 3	Overwintered terminal vegetative buds approximately 1 week prior to bud flush
S	April 18	Newly expanding shoots (average shoot elongation = 38 mm)
ST1	May 3	Shoot tips, including unexpanded leaves
ST2	June 19	Shoot tips, including unexpanded leaves
VB3	August 7	Terminal vegetative buds
VB4	October 15	Terminal vegetative buds
FB	October 15	Inflorescence buds

**Table 2:** Description of poplar genes and primers for real-time PCR.

**Name**^a^	**GenBank accession number**^b^	***Arabidopsis *homolog locus**^c^	***Arabidopsis *locus description**	**BLASTX score/ E value**	**Primer sequences (forward/reverse)**
*ACT11*	CA824001	AT3G12110	Actin 11	363/ e-101	CACACTGGAGTGATGGTTGG / ATTGGCCTTGGGGTTAAGAG
*ACT2*	BU879695	AT5G09810	Actin 2/7	320/ 5e-088	CCCATTGAGCACGGTATTGT / TACGACCACTGGCATACAGG
*CYP*	BU875027	AT2G21130	cyclophilin (CYP2)	284/ 6e-077	GGCTAATTTTGCCGATGAGA / ACGTCCATCCCTTCAACAAC
*TUA*	CA822230 CA825391	AT5G19780	tubulin alpha-3/alpha-5 chain	439/ e-130	AGGTTCTGGTTTGGGGTCTT / TTGTCCAAAAGCACAGCAAC
*TUB*	CA824237	AT4G20890	tubulin beta-9 chain	154/ 4e-038	GCACCAACTTGTTGAGAATGC / TTTCAACTGACCAGGGAACC
*UBQ*	BU879229	AT4G05050	polyubiquitin (UBQ11)	416/ e-117	GTTGATTTTTGCTGGGAAGC / GATCTTGGCCTTCACGTTGT
*UBQ-L*	BU871588	AT2G35635	ubiquitin-like (UBQ7)	291/ 4e-079	TGAGGCTTAGGGGAGGAACT / TGTAGTCGCGAGCTGTCTTG
*EIF4B-L*	CA825614	AT4G38710	similarity to eukaryotic translation initiation factor 4B	80/ 1e-015	AAAAAGGGGATTTGGGATTG / AACTTCGTCCTCGGTAGCAA
*EF1β*	BI125345	AT2G18110	elongation factor 1-beta, putative	122/ 1e-028	AAGAGGACAAGAAGGCAGCA / CTAACCGCCTTCTCCAACAC
*18S*	AF206999	18RRNA	18S ribosomal RNA	2949/ 0.0	AATTGTTGGTCTTCAACGAGGAA/ AAAGGGCAGGGACGTAGTCAA

^a^All poplar sequences except 18S are ESTs, and were named based on similarity to *Arabidopsis *proteins determined via BLASTX (Altschul et al. 1997). In most cases, the name indicates only a gene family or subfamily rather than a specific member of a gene family because partial poplar sequences and BLAST will not necessarily identify the putative *Arabidopsis *ortholog. ^b^Two accession numbers indicate that two EST sequences were used to design the primer set. ^c^Closest *Arabidopsis *homolog identified using Tair BLAST 2.0 . AGI proteins database was queried with poplar nucleotide sequences using BLASTX or in the case of 18S, *Arabidopsis *genome database with BLASTN.

**Table 3:** Summary of statistics measuring stability of gene expression

**GENE**	**MEAN**^a^	**F**^b^	**MSE-ANOVA**^c^	**CV (%)**^d^	**SLOPE**^e^	**INTERCEPT**	**R**^2^	**MS-REG**^f^	**STABILITY INDEX**^g^
***UBQ***	15.8	1.42	0.53	3.4	0.11	15.3	0.56	0.49	0.37
***TUA***	28.9	1.33	1.56	5.4	0.10	28.7	0.28	1.65	0.54
***18S***	14.0	4.72**	0.58	4.1	0.20	13.2	0.52	0.38	0.83
***ACT2***	17.3	1.29	1.25	7.2	0.16	16.6	0.57	1.14	1.16
***UBQ-L***	19.8	1.97	1.57	7.9	0.17	19.1	0.35	1.62	1.35
***EF1β***	25.1	3.76**	1.87	7.5	0.28	23.8	0.6	1.41	2.09
***TUB***	17.7	4.45*	2.7	15.3	0.37	16	0.63	1.92	5.64
***ACT11***	22.8	1.01	5.71	25.0	0.24	21.7	0.37	5.29	6.01
***EIF4B-L***	20.6	11.44**	3.22	15.6	0.52	18.2	0.68	1.79	8.13
***CYP***	19.2	7.35**	5.14	26.8	0.67	16.2	0.84	2.66	17.94

^a^Data based on analysis of C_T _values. Genes are ordered, top to bottom, from those tending to show the highest stability to those showing the lowest, based on the stability index. ^b^Approximate F-tests of variance among tissue samples tested. *, P < 0.05; **, P < 0.01. Degrees of freedom for numerator were 7 and for denominator were 40, except for 18S RNA where they were 7 and 16, respectively. ^c^MSE-ANOVA represents variance among experiments and RT-PCR reactions within experiments. 18S amplification was only included in one experiment; thus, MSE-ANOVA for 18S only represents within experiment variance. ^d^Coefficient of variation (MSE divided by mean multiplied by 100). ^e^Slope of regression of gene means (over experiments and samples within experiments) against overall means for the different samples. Intercepts and coefficient of determination (R^2^) are also given for the estimated regression lines. ^f^Mean square of deviation of means from estimated regression line (MS-reg), which estimates the degree to which genes deviate from the linear model in their level of mean expression for a particular tissue sample. ^g^Stability index is the product of CV and slope (multiplication of columns 4 and 5). Genes whose expression shows the lowest random variation within tissue samples due to variation among experiments or PCR reactions (MSE-ANOVA), and whose expression depends least in a predictable way on tissue sample (slope), are preferred as controls.
